# Clinical application of DEX/CRH test and multi-channel NIRS in patients with depression

**DOI:** 10.1186/s12993-016-0108-x

**Published:** 2016-08-31

**Authors:** Shinya Kinoshita, Tetsufumi Kanazawa, Hiroki Kikuyama, Hiroshi Yoneda

**Affiliations:** 1 Department of Neuropsychiatry, Osaka Medical College, 2-7, Daigaku-Cho, Takatsuk, Osaka 569-8686 Japan; 2 Department of Psychiatry, Shin-Abuyama Hospital, Osaka Institute of Clinical Psychiatry, Osaka, Japan

**Keywords:** DEX/CRH test, NIRS, Depression, Biomarker, HAMD

## Abstract

**Background:**

To reduce the number of patients with depression, biomarkers for clarifying psychiatric disorders are warranted. Numerous candidates have been proposed; however, near-infrared spectroscopy (NIRS) with multi-channel probes and a dexamethasone/corticotropin-releasing hormone (DEX/CRH) test are still surviving for practical demand. Thirty-one outpatients with depressed moods were analyzed using both biological tests.

**Results:**

The non-suppressors, as indicated by the DEX/CRH test, exhibited a high severity on the Hamilton Depression Scale and severe anxiety on the State Trait Anxiety Scale. In addition, a unique response was identified via NIRS in the same group suggested by the DEX/CRH assessment.

**Conclusions:**

The results obtained from these biological tests did not fit well with the category defined by operative diagnostic criteria, such as the Diagnostic and Statistical *Manual* of Mental Disorders or The International Classification of Diseases. Thus, it is critical that the utility evaluations of candidate biomarkers not be assessed by comparisons with the categorized criteria for a specific psychiatric disorder.

*Trial registration* UMIN000013214, Registered 21 February 2014

## Background

A depressed mental state is caused by non-specific mental disorders, such as a mood disorder, schizophrenia, substance abuse, a personality disorder, or nearly every psychiatric condition. If it is more prolonged than expected, several types of costs related to individuals with depressed moods will increase. A recent analysis indicated a 21.5 % cost increment, $173.2 billion (2005) to $210.5 billion (2010), within 5 years, and this cost will be further increased [9]. Another analysis with regard to the global DALY (disability-adjusted life year) indicated that the cost associated with depression would represent the primary cost of all disorders, including physical disorders, in 2030, and it ranked 3rd in 2004 (http://www.who.int/healthinfo/global_burden_disease/GBD_report_2004update_part4.pdf). These analyses have provided warnings regarding the importance of depression prevention. In addition, to perform effective treatment, a precise assessment based on scientific data comprises a key factor.

Evidence regarding the relationship between depression severity and blood flow measurements scaled by Near-Infrared Spectroscopy (NIRS) is accumulating [30, 36, 41]. NIRS comprises a non-invasive imaging device that uses multiple channels to visualize brain activity. It has been developed as a result of the demand to consider a differential diagnosis in patients with depressed moods despite the administration of antidepressant agents for an extended period. A reliable biological marker is required to identify a specific psychiatric disorder because diagnosis in psychiatry currently relies on expressed symptoms or spoken words. NIRS comprises a device that applies knowledge of neuroscience through the assessment of blood flow in the brain; however, another potential candidate for a reliable biomarker depends on an endocrine imbalance. Particularly in the assessment of disturbed regulation of the hypothalamic-pituitary-adrenocortical (HPA) system, dexamethasone/corticotropin-releasing hormone (DEX/CRH) has been extensively investigated with a view towards the differential diagnosis of depression prior to the development of NIRS. Originally, the dexamethasone suppression test (DST) was used to diagnosis Cushing’s syndrome [22]; this endocrine reaction has also been implemented for the diagnosis of endogenous depression. The current standard method is a DEX/CRH test in combination with the administration of CRH, which has been reported to increase the sensitivity from 60 to 80 % for the classic type of depression [12, 13]. There is an unavoidable burden regarding the application of this method for practical usage; however, these two biological methods have been considerably implemented in previous decades for investigating the biomarkers of depression.

In the current study, we investigated patients with a depressed mental state. Following a diagnosis according to the DSM-IV or psychological assessments, two tests using both endocrine (DEX/CRH) and neuroimaging (NIRS) assessments were applied. The aim of the current work was to identify the relationship between the psychiatric diagnosis based on the expressed symptoms and the assessment of two biomarkers.

## Methods

### Subjects

The subjects comprised 31 outpatients (15 men and 16 women; mean age: 44.2 years, SD: 12.2 years) with a depressive state, who were recruited from patients who attended the Neuropsychiatry Department of Osaka Medical College. The demographic details of the psychiatric diagnoses are shown in Table 1. We have adopted structured clinical interview for DSM-IV TR (SCID) for clinical assessment [7].

**Table 1:** Psychiatric diagnoses assessed by the DSM-IV TR for study patients (n = 31)

SCID	Major depressive disorder	Somatoform disorder	Panic disorder	Bipolar II disorder	Schizophrenia	Obsessive–compusive disorder	Bipolar I disorder	Dysthymic disorder	Psychotic disorder
DSM code	296.2 and 296.3	300.82	300.21	296.89	295.3	300.3	296.53	300.4	298.9
Number	17	2	2	1	1	1	3	1	3

The severity of the index depressive episode was assessed with the 21-item version of the Hamilton depression rating scale (HAMD) [10, 15], on the admission day (DEX/CRH on the 2nd day and NIRS on the 3rd day). Enrolled patients (n = 31) were not strictly limited along with the HAMD score because all of the participants had the difficulty to live their life. Regarding the self-evaluation tool for anxiety, the STAI, the State-Trait Anxiety Inventory [26, 34], was administered to the participants on admission day. The majority of the patients were medicated with antidepressants; however, we did not control for the class of antidepressant medication. In addition, we assessed the psychiatric disorders of patients along with the SCID method; therefore, psychiatric comorbidities were not controlled.

### DEX/CRH test

The DEX/CRH test was conducted according to the method described by Zobel et al. [42]. The subjects were pretreated with an oral dose of 1.5 mg of DEX at 2200 h on the 1st admission day. On the next day, a vein was cannulated at 1400 h to collect blood at 1430, 1500, 1530, 1545, and 1600 h via an intravenous catheter. Human CRH (100 μg) was intravenously administered at 1400 h immediately after the initial blood collection. The plasma concentrations of ACTH and cortisol were measured via radioimmunoassay at SRL Corporation (Tokyo, Japan). The detection limits for ACTH and cortisol were 5.0 pg/ml and 1.0 μg/dl, respectively.

The definition of the subtypes of the cortisol suppression pattern followed previously described criteria [19, 14] and included incomplete-suppressors (DEX/CRH-cortisol ≥5 μg/dl, moderate-suppressors (1 μg/dl ≤ DEX/CRH-cortisol <5 μg/dl), and enhanced-suppressors (DEX/CRH-cortisol <1 μg/dl). We also included a non-suppressor group with cortisol >5 μg/dl at the test onset to reduce the number of incomplete suppressors (onset-cortisol <5 μg/dl).

### NIRS measurement

The NIRS measurements were performed using a 22-channel ETG-4000 Optical Topography System (Hitachi Medical Corporation, Tokyo, Japan). This machine used two sets of wavelengths of near-infrared light (695 and 830 nm, respectively) to identify differences in the absorption spectrum, which thus enabled the measurement of oxy-Hb and deoxy-Hb [23]. Seventeen emitter probes and 16 detector probes were plugged into a 3 × 11 array. The distance between the pair of emission and detector probes was 3.0 cm; the measuring area between each pair of detector probes was defined as a “channel.” Probes were placed on the frontal region of the participant. The lowest probes were positioned along the Fp1–Fp2 line in accordance with the international 10–20 system used in electroencephalography.

### Activation task

Changes in the hemoglobin concentration were measured during the verbal fluency task [37]. The cognitive activation task was structured to include a 30-s pre-task period, a 60-s task period, and a 70-s post-task period. For the pre- and post-task baseline periods, the participants were instructed to consecutively repeat five Japanese vowels (a, i, u, e, o) aloud. During the task periods, they were instructed to generate as many Japanese words as possible that began with a designated syllable. The initial syllables were presented in counterbalanced order among the participants with each syllable changing every 20 s (0–20 s: /to/, /na/, /a/; 20–40 s:/se/, /i/, /ki/; 40–60 s: /o/, /ta/, /ha/) during the 60-s task period.

### Measurement environment

Each participant was seated in a comfortable chair and instructed to remain still to prevent movement artifacts; specifically, head movements, strong biting, or unnecessary eyebrow movements were minimized during the NIRS measurements. The data that clearly contained motion artifacts, based on both our observations and NIRS recordings, were excluded from further analyses.

### Statistical analysis

All statistical analyses were performed with JMP Pro^®^ software (Ver. 11.0, SAS Institute Japan Ltd., Tokyo, Japan). Intergroup comparisons were conducted for HAMD, STAI scores and the gained scores from NIRS instrument according to the Kruskal–Wallis test. A value of p < 0.05 was considered statistically significant.

## Results

According to the previously described criteria for the division of the DEX/CRH response, the numbers of participants in the four groups divided by the cortisol reaction are indicated in Table 2.

**Table 2:** Number of patients with depression divided by the DEX/CRH test (n = 31)

	DEX/CRH-cortisol	Onset cortisol	Male	Female	Total
Enhanced-suppressors	<1 μg/dl		2	5	7
Moderate-suppressors	1 μg/dl ≤ <5 μg/dl		2	8	10
Incomplete-suppressors	≥5 μg/dl	Onset cortisol <5 μg/dl	8	3	11
Non-suppressors	≥5 μg/dl	Onset cortisol ≥5 μg/dl	3	0	3

**Fig. 1:**
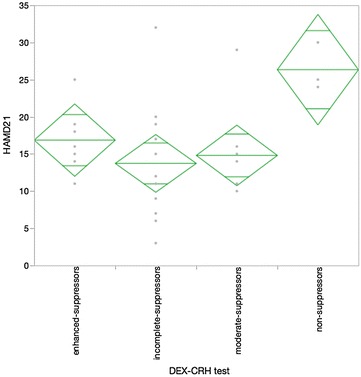
*Diamond plot* of the HAMD 21 assessment and DEX–CRH test (n = 31). Kruskal–Wallis analysis Chi square 7.37, p = 0.06. Post hoc analysis non-suppressors, 26.3 ± 3.2, vs incomplete suppressors, 13.7 ± 8.2, p = 0.04, vs moderate suppressors, 14.8 ± 5.8, p = 0.03

**Fig. 2:**
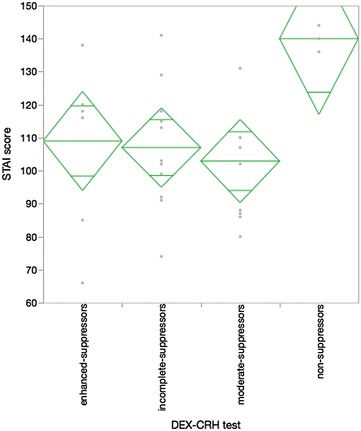
*Diamond plot* of the STAI assessment and DEX–CRH test (n = 31). Kruskal–Wallis analysis Chi square 7.58, p = 0.06. Post hoc analysis non-suppressors, 140 ± 4.0, vs incomplete suppressors, 107 ± 18.8, p = 0.03, vs moderate suppressors, 103 ± 18.1, p = 0.01, vs enhanced suppressors, 109 ± 24.6, p = 0.04

**Fig. 3:**
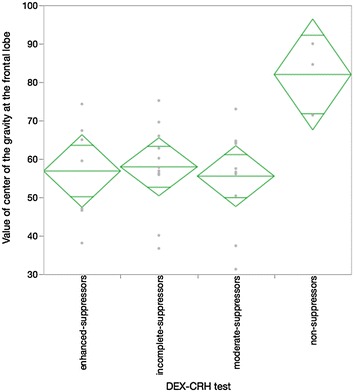
*Diamond plot* of the NIRS assessment (value of the center of gravity at the frontal lobe) and DEX–CRH test (n = 31). Kruskal–Wallis analysis Chi square 7.02, p = 0.07. Post hoc analysis non-suppressors, 82.0 ± 9.6, vs incomplete suppressors, 58.0 ± 11.4, p = 0.02, vs moderate suppressors, 55.5 ± 12.8, p = 0.02, vs enhanced suppressors, 56.9 ± 13.2, p = 0.04

First, the association between the reaction in the DEX/CRH test and the severity of depression determined by the HAMD assessment was investigated. The HAMD was recorded by another clinician in a blind manner. There was a difference in the mean value of the HAMD score between the non-suppressors and the other two main groups, incomplete suppressors and moderate suppressors (Fig. 1 Kruskal–Wallis analysis Chi square 7.37, p = 0.06). Second, the STAI, which is a self-evaluation tool for anxiety, significantly represented the characteristic psychological feature of the non-suppressor group compared with the other three groups (Fig. 2 Kruskal–Wallis analysis Chi square 7.58, p = 0.06). Moreover, we compared the results of the NIRS assessment with the reaction of the DEX/CRH test. Similar to the HAMD assessment, the NIRS recording was performed by a lab technician in a blind manner. The results indicated that the values of the center of gravity at the frontal lobe in the non-suppressor group, which comprised the representative values in NIRS, were significantly different compared with the other three groups by post hoc analysis (Fig. 3 Kruskal–Wallis analysis Chi square 7.02, p = 0.07). No significant age difference between the groups that were defined by the two biological methods was found (data not shown).

## Discussion

The current sample size is relatively small to provide a definitive conclusion; however, our current work has an advantage in which we employed cases regardless of the diagnosis of psychiatric disorders. Therefore, there is the potential that the novel scientific findings identified from our sample are consistent across diagnoses based on the operative diagnostic criteria, such as the DSM. In addition, this is the first report to assess confirmed cases with depressed mood by two representative assessment methods for depression.

Regarding the DEX/CRH test, it has been reported that 60–80 % of patients with depression exhibited an increased cortisol response prior to the initiation of treatment [18, 42]. It is partly because all participants in this study received medical treatment prior to the assessment, whereas only 11 % (3/31) of the participants in this study exhibited an increase cortisol response (non-suppressors). Moreover, most previous studies utilizing DEX/CRH test have been based on data obtained from inpatients [18, 20], which implies a clinical subgroup with severe symptoms, whereas our current sample consisted of a depression severity at the outpatient level. Moreover, the group that enrolled the largest sample size did not include data for individuals with a HAMD less than 15 [18], whereas the average HAMD score was 15.6 ± 7.5 in our sample. This difference has a significant impact on the interpretation of our data. The percentage of non-suppressors was not high because of the enrolled mild subtype of depression; however, every non-suppressor patient exhibited a high score on the HAMD assessment (average score of HAMD: 27.8) despite inconsistencies in the psychiatric disorders across three patients (two patients: 296.23 major depressive disorder, and one patient: 300.3 obsessive compulsive disorder). Thus, the DEX/CRH approach should again be considered a potentially useful biomarker for dividing the subclasses of depression.

With regard to psychological aspects assessed by STAI, the Kruskal–Wallis analysis indicated there was no significant difference for the divided subgroups regarding the DEX/CRH test; however, a trend was identified particularly for the non-suppressor group (Chi square 7.58, p = 0.06). Anxiety is a non-specific feature of psychiatric disorders. Therefore, the severity of anxiety is not fundamentally considered to be scaled by a biological method. Although the scale is simply based on a self-evaluation scale, our results indicated the potential finding that anxiety across psychiatric disorders, including depression, is represented by a biological scale, such as DEX/CRH. Anxiety-related stimuli cause a systemic response through neural circuits centered on the amygdala, which enhances the increment of cortisol [38]. Therefore, an association between the altered DEX/CRH result and the anxiety scale is reasonable. A previous study demonstrated that individuals with depression and comorbid anxiety (n = 18) related disorders exhibited a significantly lower cortisol level compared with pure depression patients (n = 36) [40]. The non-suppressor group, which showed higher anxiety, more severe depressive symptoms and a distinguished response in the DEX/CRH, should be regarded as one entity in terms of different biological and psychological reactions. Thus, larger sample sizes, especially for the non-suppressor group, are necessary to clarify the association between the STAI scale and DEX/CRH results.

Accumulating evidence supports the relationship between NIRS and the diagnosis of psychiatric disorders [21, 24, 36]. Thus, NIRS is one of the most attractive tools for adopting a biological assessment for clinical practice. One reason is that the device requires only 10–15 min for each individual if VFT is adopted for the assessment. NIRS is sufficiently easy to adopt, even for adolescents, for the evaluation of psychiatric disorders, such as depression, bipolar disorder, and schizophrenia. General interpretation on VFT is a neurocognitive battery for assessing verbal ability or executive control ability. This battery is widely used for assessing these abilities of patients after stroke, patients with Alzheimer’s disease or Parkinson’s disease [33]. In the current study, we have utilized this battery mainly for recording the measurements of oxy-Hb in the brain, therefore the task performance (the number of words) on each individual were not recorded. The comparison of the performance on VFT between each groups will be of interest. Future work should focus on the task performance across various psychiatric disorders. One of the critical issues regarding the NIRS device is that insufficient data are obtained from the perspective of neuroscience. In an analysis of a larger sample than the current work, our group recently demonstrated that the severity of depression scaled by the HAMD was negatively associated with the integral value of the blood flow at the frontal lobe (n = 43) [16]. This finding was characterized by the point that the assessment was not limited to depressed patients who satisfied the criteria defined by the DSM or ICD. Moreover, several biomarkers, such as DEX/CRH, NIRS and other factors, cannot guarantee the specificity of psychiatric disorders defined by operational diagnostic criteria. The current findings indicated that individuals with a Non-Suppression reaction in the DEX/CRH test exhibited a fairly increased value of the center of gravity at the frontal lobe. A general interpretation regarding the increased value of the center of gravity implies the existence of bipolarity [36]. In specific areas such as the left inferior frontal lobe, [oxy-Hb] was decreased in patients with panic disorders [27, 28]. Although the current work did not focus on a specific channel of NIRS, future research should analyze the data for a specific channel by assessing a larger sample series. Thus, a larger sample size is critical to validate the current work to determine the unknown mechanism of endocrine imbalance and brain response. In addition, the biological data from the mentally healthy controls will serve as a useful reference, although the current design has not been adopted to collect them. Moreover, the length of the duration of mental illness has a possible effect on the result from the biological assessment. This should be considered in future studies.

Biological assessments embedded in the diagnosis of specific psychiatric disorders are proceeding. Candidate methods have been evaluated using the concordance rate for diagnosis defined by the operative criteria to date; however, a universally accepted biomarker has not been identified. Despite substantial efforts to identify a biological marker, the methodological approach for objective evaluation is incorrect in terms of the fact that the concordance rate between biological assessments and psychiatric diagnoses based on the artificial operative system is not sufficient to satisfy practical demands. For example, a genetic approach has indicated considerable overlap between schizophrenia and bipolar disorder [6, 29]; therefore, the current understanding regarding these disorders has been modified to indicate that the two disorders are related diseases in terms of the genetic aspects [31]. Moreover, recent reports have indicated considerable genetic overlap across several types of psychiatric disorders [4, 8, 11, 25]. According to a recent perspective based on genetic analyses, Craddock and his group have advocated the theory that psychiatric disorders are considered a spectrum [3]. It is true that a decreased frontal lobe is present in the brains of individuals with schizophrenia and bipolar disorder, as well as depression [5, 35]. Regarding therapeutic agents, antidepressants are effective for the improvement of symptoms in schizophrenia and bipolar disorders [32, 39]. Alternatively, antipsychotic agents are used for the treatment of depression [2]. Based on the traditional diagnostic category, it is impossible to explain the differences between psychiatric disorders by genetic or morphological factors or the responsiveness to therapeutic drugs. Our current finding indicates a trend of correlation between the severity of depression and the response to the DEX/CRH test; however, the diagnostic categories did not sufficiently fit the biological assessment. Moreover, our findings also suggest that the current diagnostic criteria are not valid for the assessment of biological markers in psychiatric disorders. Thus, the diagnostic criteria must be updated based on the contributions of biological assessments, such as DEX/CRH or NIRS [1, 17].

## Abbreviations

DEX/CRH: dexamethasone/corticotropin-releasing hormone
NIRS: near-infrared spectroscopy
ACTH: adrenocorticotropic hormone
HAMD: Hamilton rating scale for depression
STAI: state-trait anxiety inventory
